# Society Meetings

**Published:** 1900-10

**Authors:** 


### SOCIETY MEETINGS.

The Esculapian Club, of Buffalo, was entertained by Dr. A. F. Colton, at his home, 41 Otis Place, Thursday evening, September 20, 1900. Dr. A. W. Bayliss was the essayist of the evening, and his subject, which was interestingly handled, was The Uses of Electricity in General Practice.

---

The Tri-State Medical Society will hold its twelfth annual meeting at Chattanooga, Thursday, Friday, Saturday, October 11, 12 and 13, 1900, just after the meeting of the Mississippi Valley Medical Association, at Asheville, N. C. A rate of one fare for the round trip will be in force on the occasion of the reunion of the Army of the Cumberland and the Spanish-American war veterans. The secretary is Dr. Frank Trester Smith, Chattanooga, Tenn., who should be addressed on any subject relating to the meeting.

---

The Medical Association of Central New York will hold its thirty-third annual meeting at Rochester, Tuesday, October 16, 1900. All who desire to read papers or present cases may do so by communicating with the president, Dr. John Gerin, Auburn, N. Y.; Dr. C. A. Van der Beek, Rochester, N. Y., or the Journal office. The association publishes an annual volume of transactions and ranks with the best medical societies in the state.

---

The American Association of Obstetricians and Gynecologists held its thirteenth annual meeting at Louisville, Ky., Tuesday, Wednesday and Thursday, September 18, 19 and 20, 1900, under the presidency of Dr. Rufus Bartlett Hall, of Cincinnati, O.

The sessions were largely attended by Fellows of the Association as well as visitors, and the meeting was pronounced by all as one of the most successful, scientifically and socially, in the history of the Association.

The program was an unusual one, embracing almost every phase of gynecologic surgery, as well as many other subjects of interest to special workers in obstetrics, gynecology and abdominal surgery. The debates were full of spirit and extremely instructive. Members were present from the four quarters of the domain—from Massachusetts and Texas; from Arkansas and Canada; from Alabama and Wisconsin. The American character of this Association was thus fully emphasised, and it was never in a more flourishing condition than at present.

The social amenities were of the first order, beginning with luncheons during the noon recess on the first day, continuing during the two succeeding days, and reaching a climax at the annual dinner, served at the Galt House. At this function after the viands were liberally discussed, both song and speech served to enliven an hour or two over the coffee. The songs of Miss Anita Muldoon will ever remain a sweet memory with every one who heard her delightful voice.

The next meeting will be held at Cleveland, O., in September, 1901. Officers were elected as follows: President, Dr. W. E. B. Davis, of Birmingham, Ala.; vice-presidents, Dr. Edwin Walker, of Evansville, Ind., and Dr. A. Goldspohn, of Chicago; secretary, Dr. William Warren Potter, of Buffalo, N.Y., and treasurer, Dr. X. O. Werder, of Pittsburg, Pa.

---

THE Buffalo Academy of Medicine held meetings during the month of September, 1900, as follows:

*Section on Surgery.*—Tuesday evening, September 4th. Program: Angioma, Vertner Kenerson; Resection of joints, with cases, Herman Mynter.

*Section on Medicine.*—Tuesday evening, September 11th. Program: Clinical quantitative estimation of proteid digestion in the stomach, with demonstrations, A. L. Benedict.

*Section on Ophthalmology, Otology, Rhinology and Laryngology.*—Monday evening, September 17th. Program: Eye-strain or gastric reflex? A. L. Benedict; discussion opened by Drs. Stockton, A. A. Jones, E. Starr and Bennett.

*Section on Pathology.*—Tuesday evening, September 18th. Program: Blood examinations, H. G. Matzinger; discussion opened by Drs. Julius Ullman and Albert E. Woehnert.

*Section on Obstetrics.*—Tuesday evening, September 25th. Program: A few glimpses, including medical ones, of lands across the sea, Francis E. Fronczak. Obstetric view, from a gynecological standpoint, Charles E. Congdon.

---

THE American Public Health Association will hold its twenty-eighth annual meeting at Indianapolis, Ind., Tuesday, Wednesday, Thursday and Friday, October 22-25, 1900.

The Southern Surgical and Gynecological Association will hold its thirteenth annual meeting in Atlanta, November 13-15, 1900, under the presidency of Dr. A. M. Caitledge, of Louisville. The indications are favorable for a successful session. Members of the medical profession are cordially invited to attend. The secretary, Dr. W. E. B. Davis, of Birmingham, Ala., should be addressed on all questions relating to the meeting.

